# Race Guides Attention in Visual Search

**DOI:** 10.1371/journal.pone.0149158

**Published:** 2016-02-22

**Authors:** Marte Otten

**Affiliations:** 1 University of Amsterdam, Amsterdam, The Netherlands; 2 University of Sussex, Brighton, United Kingdom; University of Bologna, ITALY

## Abstract

It is known that faces are rapidly and even unconsciously categorized into social groups (black vs. white, male vs. female). Here, I test whether preferences for specific social groups guide attention, using a visual search paradigm. In Experiment 1 participants searched displays of neutral faces for an angry or frightened target face. Black target faces were detected more efficiently than white targets, indicating that black faces attracted more attention. Experiment 2 showed that attention differences between black and white faces were correlated with individual differences in automatic race preference. In Experiment 3, using happy target faces, the attentional preference for black over white faces was eliminated. Taken together, these results suggest that automatic preferences for social groups guide attention to individuals from negatively valenced groups, when people are searching for a negative emotion such as anger or fear.

## Introduction

Almost without exception, natural, socially relevant visual scenes contain many objects. Too many, it seems, for the visual system to process in parallel, even for simple scenes existing of inanimate objects [1,2]. As a result, the visual system needs to select those stimuli that are most relevant as its focus of attention, to be further processed and encoded.

In the study of attention, most research has focused on determining which basic psychophysical features automatically capture our [3,4]. However, in situations where stimuli also have rich social texture and relevance, it becomes equally important to learn whether higher order social processes like beliefs, attitudes, and intergroup preferences influence which items capture attention. Even though the mid-century “New Look” movement already made a strong case that social processes critically change basic cognitive functioning (see for example [5]), we still know very little about this important interaction between higher order social processes and basic cognition. In a notable exception, a number of recent studies show that both internal emotional states and traits can influence the way people attend to emotionally relevant stimuli (see [6] for a review). For example, Öhman, Flykt and Esteves [7] showed that for those who are fearful of spiders, images of spiders draw far more attention than for non-phobics.

A similar attentional process could play a role in interpersonal attention as well. Trawalter and colleagues [8] rapidly flashed a black and a white face side by side on a computer screen, after which a dot appeared behind one of the two faces. Participants were faster to respond to a dot that appeared behind the black face compared to the white face, suggesting that the black face attracted attention over the white face. Donders, Correll and Wittenbrink [9], using similar dot-probe design, found this attentional preference for black faces only in participants who have a strong implicit association between black and danger. Electrophysiological research also suggests that black faces evoke stronger attentional processing than white faces, as indicated by a larger N1/P2 complex for black compared to white faces [10–13]. Taken together, these behavioral and neural findings suggest that cues about a person’s racial background, combined with internal racial stereotypes, draw attention to specific faces, and can also guide attention when searching for a specific target, at least in situations where attention is divided between two people.

However, in everyday life the visual and social environment is not limited to two people, and guiding attention is not a simple dichotomous choice. To test whether race and racial attitudes can guide attention in crowded visual environments one can employed a visual search paradigm [14], which allows the creation of a social environment consisting of multiple social actors who vary in racial composition. In a visual search paradigm participants are shown a display with multiple items (i.e. faces), and are asked to identify the one target that has a unique feature (i.e. the one angry face in a display of neutral faces) as quickly as possible. By analyzing search slopes across increasing numbers of faces on display (i.e. set size), such a design can test whether search for some targets is more efficient than others, and thus whether certain faces stand out more. The gradient of a search slope indicates the degree to which reaction times lengthen with an increasing set size [15]. If a target draws more attention than the distractors, search will be more efficient, producing relatively flat or shallower slopes. If, on the other hand, the target does not stand out compared to the distractors, each item in the display will need to be scrutinized, leading to an increase in reaction times for each distractor that is added to the display.

There are two studies suggesting that black faces indeed attract attention in displays containing multiple face distractors. Levin, in Experiment 5 & 6 [16] and Chiao and colleagues [17] asked participants to search for a black target face among white distractors, or a white target face among black distractors. Search slopes were smaller when participants were searching for a black face compared to a white face, which suggests that black faces attract more attention than white faces. However, in both experiments participants were explicitly instructed to search for a black or white face. This instruction could have influenced the observed behavioral patterns, either because participants changed their search strategies depending on the instruction, or because the explicit race-referenced instructions activated explicit or implicit associations with the search target, which in turn influenced search patterns.

In the present study, participants were not asked to search for faces with specific racial features. Instead, they were asked to find faces with specific emotional features (anger, fear or happiness). As such, even though the race of the target face varied between black and white, the race of the face was actually irrelevant for the search goal of the participant. Therefore, the current design allowed us to explore whether race influences visual attention when the search goal is completely unrelated to race. If black faces indeed automatically draw more attention than white faces, the emotional target face will be identified more efficiently when the face is black rather than white. On the other hand, if attention is not guided by race when race is not a relevant factor within the search process, black and white targets should be identified equally efficiently, showing comparable search slopes. Here I employ the visual search task to test if race and automatic race preferences guide attention within our immediate social environment.

## Experiment 1

Many (white) Americans still have more automatic negative attitudes toward black Americans. Will such negative associations orient attention selectively to specific faces? Here I test whether the search for a negative emotion will orient the attention of the perceiver spontaneously toward those social cues that are “prepared” by virtue of prior negative social meaning, i.e. black faces compared to white faces.

Participants searched for an emotional (angry or frightened) male face in a display of neutral faces. Anger and fear are both negative emotions, and could therefore be relevant for the participant considering the overall negative implicit associations with black men [18], although only anger is associated with the meme of the angry black man [19]. As explained above, I measured search efficiency (i.e. the steepness of the search slope, [14,20]) to test whether attention is drawn to black faces over white faces. If black faces attract more attention than white faces, the relative increase in reaction time when the display becomes more crowded is smaller for black than for white targets. I thus expected the search slope (reaction time_set size8_ –reaction time_set size4_)/ 4) to be smaller if the emotional target was black than if the emotional target was white.

It is important to note that (in contrast to search slopes) simple reaction times can also be influenced by other stages of perceiving and responding to a stimulus, such as differences in processing speed, increases in holistic face processing, response preparation, memory encoding and retrieval, etc. Simple differences in reaction times (as opposed to differences in search slopes) are thus not necessarily due to differences in attention. I will therefore focus on the search slopes (i.e. the effect of a set size increase), and not on differences in simple reaction times to black and white faces.

### Methods

#### Participants

I tested 76 participants from the Harvard University Subject Pool for payment or course credit. Of these 76 participants, 52 were women. The age of the participants ranged from 18 to 48 years, with an average of 20.5 years. 43 participants were European Americans, 24 were Asian Americans, 4 were African American, 3 were Hispanic Americans, 1 was Native American and 1 participant did not answer this question. All participants read and signed a written informed consent before taking part in the experiment. The entire experiment was approved by the Committee of the Use of Human Subjects (CUHS), the Institutional Review Board of Harvard University

#### Procedure

**Fig 1:**
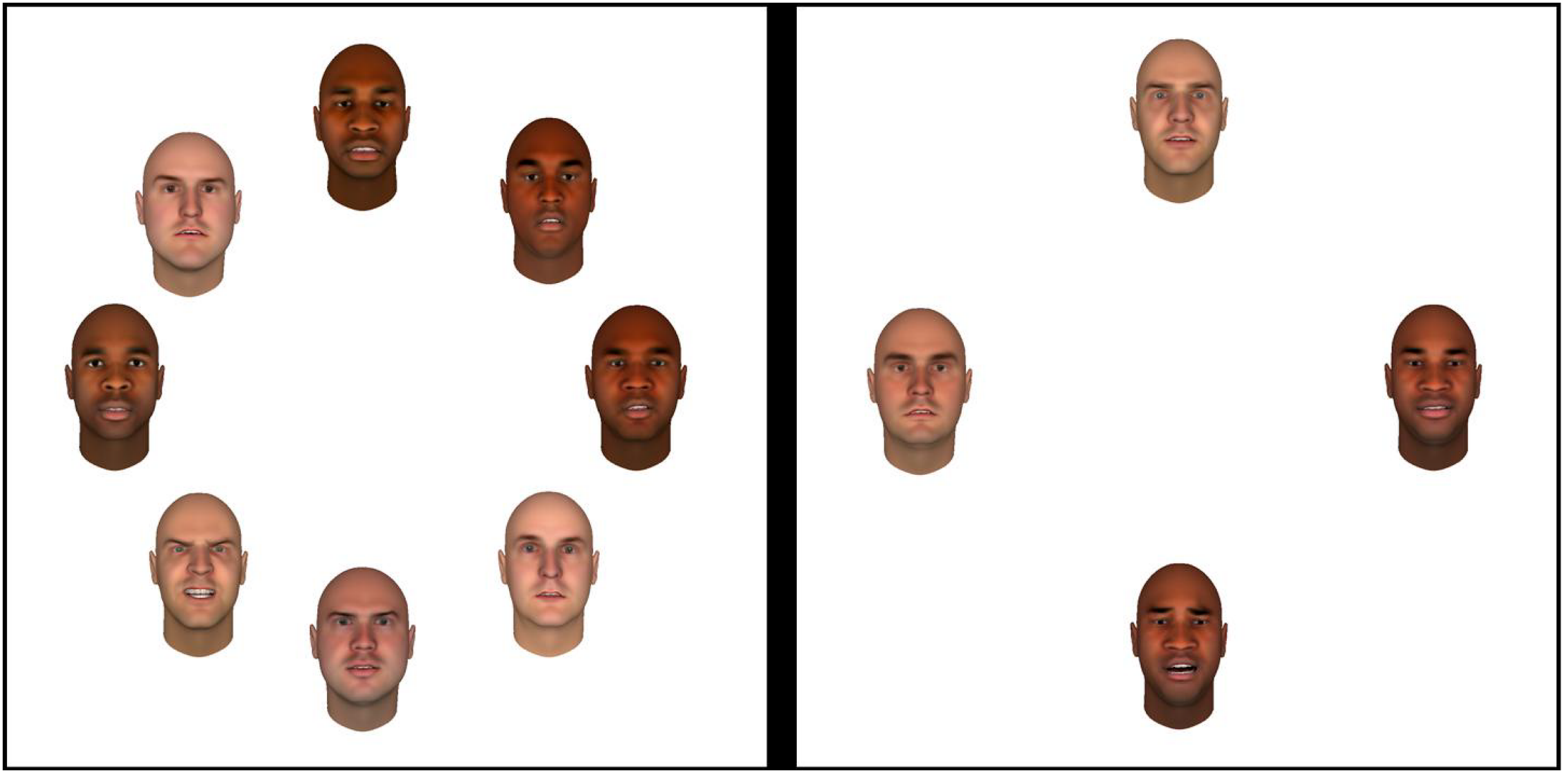
Two examples of search displays, with 4 or 8 faces. Participants were instructed to find the face with the emotional expression (angry in one half of the blocks, frightened in the other half). The display on the left contains an angry white target-face, and the display on the right contains a frightened black target-face.

Participants were instructed that they would be viewing displays that in some cases would contain an emotional face that was either angry or frightened. In other cases all the faces would have neutral expressions. Their task was to detect the presence of the emotional face (the target) within a display of faces with a neutral expression (the distractors). The search displays always consisted of 4 or 8 different faces, presented on an imaginary circle with an 8 degrees visual angle radius from the center of the computer screen. All stimuli were 1.6 degrees visual angle in width and 2.5 degrees visual angle in height, and they were displayed within equal distance of each other and the center of the screen. In each display, half of the faces were black and the other half were white. Fig 1 shows two examples of such search displays.

Participants started the experiment with instructions about the task, followed by 5 practice trials which were not included in our analyses. Subsequently they completed 256 experimental trials, divided over 8 blocks of 32 trials. In 128 of the 256 trials, an emotional target face was present, which could be black (50% of the target-present trials) or white. In addition, the emotion displayed by the target face varied within the experiment. In 4 consecutive blocks participants were instructed to search for an angry target, and in the remaining 4 consecutive blocks they searched for a frightened target (sequence was counterbalanced over participants).

Blocks were separated by breaks which lasted a maximum of 2 minutes, but participants could start the next block earlier if they wanted by pressing a button. Each trial consisted of a fixation point which was presented for 800 ms, followed by the search display. The display remained on the screen until the subject pressed a response key. To respond whether or not a target was present, participants pressed the ‘A’ key (left hand) or the ‘L’ key (right hand), the response mapping was counterbalanced over participants. After each response, participants received feedback which included their response time and whether their response was correct or not. For correct responses with a reaction time longer than 3000 ms participants received feedback that their response had been correct, but too slow. This response-deadline of 3 seconds was chosen since from pretests I observed that search times for 8 picture target-absent displays (the slowest condition) was approximately 2 seconds on average.

The experiment was programmed in Matlab, using the PsychToolbox, running on a PC. The stimuli were displayed on a fast TFT monitor placed approximately 55 centimeters from the participant.

In total, the experiment used 60 different pictures (20 distractors and 20 angry targets and 20 frightened targets). Target- and distractor-faces were generated using Facegen software. I generated 10 different white and 10 different black identities using the ‘Generate’ function available within the Facegen software package. The settings for all identities were as follows; Gender: male, Age: 30, Caricature: the average, Asymmetry: symmetrical. For black identities the Race settings were set to African in all race categories. For white identities the Race settings were set to European in all race categories. For each of the resulting 20 identities I created 3 pictures with different facial expressions using the ‘Morph’ function available within the Facegen software package: one face with a neutral expression (all modifiers set to 0), one with an angry expression (modifier Anger at 0.8) and one with a frightened expression (modifier Fear at 0.8).

The Facegen faces were rated for emotional intensity by an independent sample of particpants (10 participants, mean age 43, 3 women). All faces were rated on a 100 point scale, with 0 indicating ‘totally neutral’ and 100 indicating ‘extremely emotional’. Overall, white and black faces were rated as equally emotional, for both anger (white: 53.2 (16), black: 53.1 (16)) and fear (white: 54.4(13), black: 49.6 (15.9)).

#### Analysis

Only the trials in which participants correctly identified the presence of a target were used for the analysis. Outliers were removed according to the following procedure: For each participant and condition, the mean and standard deviation of the response times were calculated. Reaction times that were larger than the mean +/- 2*standard deviation were removed from the analysis. As a result of removing outliers and trials in which participants missed the presence of a target, 16% of the data were excluded from the analysis (9% as a result of incorrect responses, 5% following outlier correction). The remaining data were subjected to an ANOVA with Set Size (4/8), Race of Target (black/white) and Emotion of Target (anger/ fear) as within-subjects factors. The results are reported for the full sample of participants (76 participants), as well as for a smaller sample that excludes the African American participants (72 participants). The statistical tests for the full sample are always presented first, with the results from the smaller sample presented in parentheses.

### Results and discussion

Overall, participants were faster to detect angry faces than frightened faces (Target Emotion, *F*(1,75) = 18.46, *p* < .0001, partial η^2^ = .20 (*F*(1,71) = 17.88, *p* < .001, partial η^2^ = .20)). Although over the two different search goals black targets were not detected faster than white targets (Race, *F*(1, 75) = .01, *p* = .90, partial η^2^ < .001 (*F*(1,71) = .13, *p* = .72, partial η^2^ < .01)), the interaction between Race and Target Emotion was significant (*F*(1, 75) = 8.3, *p* < .01, partial η^2^ = .10 (*F*(1,71) = 7.41, *p* = .008, partial η^2^ = .10)): black targets were detected faster than white targets when subjects searched for angry faces (*t*(75) = 2.13, *p* = .04, Cohen’s *d* = .50 (*t*(71) = 1.79, *p* = .08, Cohen’s *d* = .42)), whereas frightened white targets were found somewhat, although not significantly, faster than frightened black targets (*t*(75) = 1.45, *p* = .16, Cohen’s *d* = .32(*t*(71) = 1.51, *p* = .13, Cohen’s *d* = .36)).

However, as I discussed above, faster response times can be caused by many factors (i.e. differences in response preparation or perceptual processes, etc.). Since I are specifically interested in whether black faces attract more attention than white faces, the most relevant results are signaled by interactions with set size, which indicate differences in search efficiency. When a specific target draws more attention, adding more faces to the display will not influence search times very much, leading to less steep search slopes.

**Fig 2:**
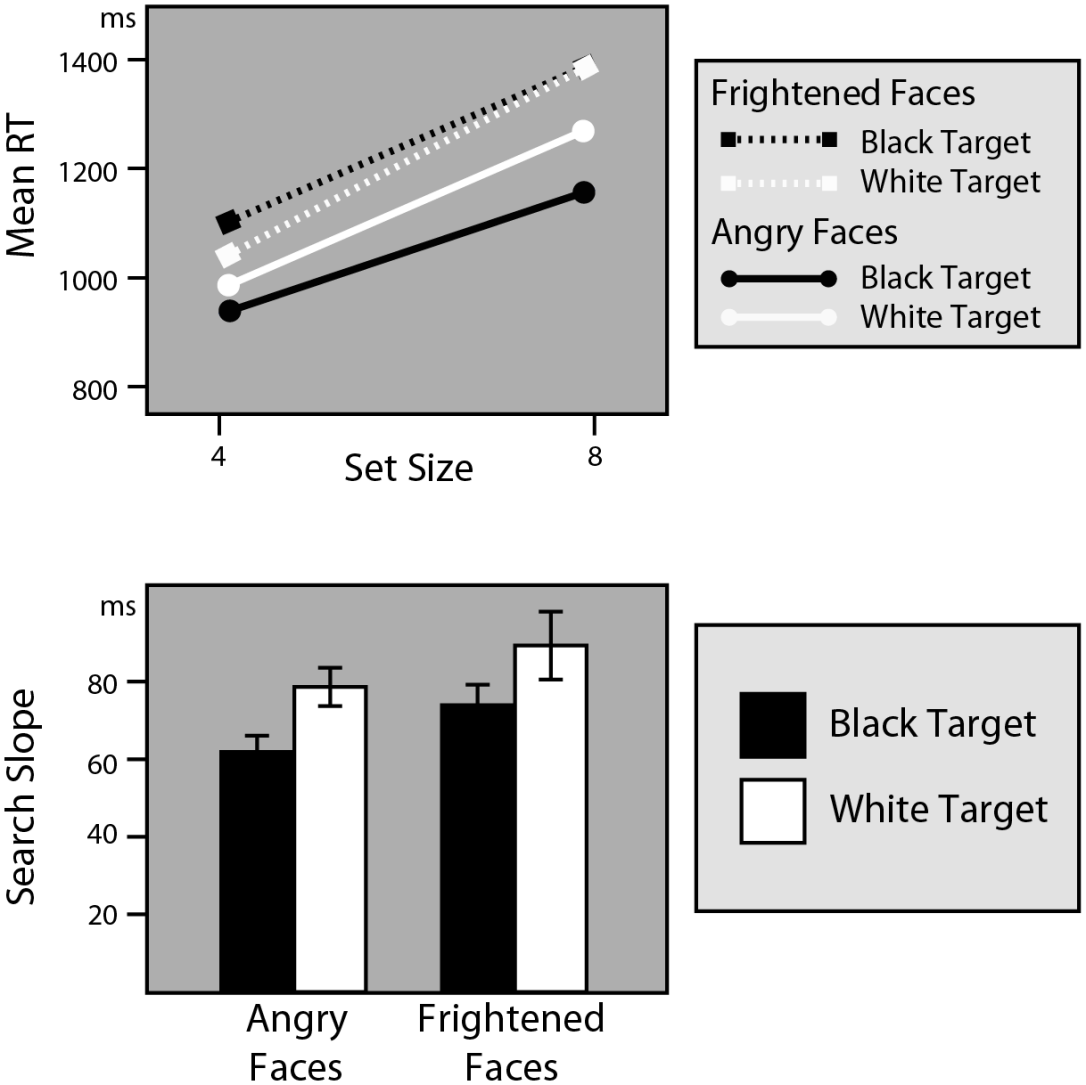
Results of Experiment 1. The top panel shows the search functions for black and white, and angry and frightened faces. The gradient of each function indicates the search efficiency, with shallow functions (lower search slopes) indicating more efficient search. Bottom panel depicts the search slopes for each of the four conditions, indicating that black faces draw more attention than white faces.

The upper panel of Fig 2 shows that it took participants significantly longer to find any of the targets in a display with 8 faces than in a display with 4 faces (Set Size, *F*(1, 75) = 315.91, *p* < .0001, partial η^2^ = .81 (*F*(1,71) = 303.89, *p* < .0001, partial η^2^ = .81)). The lower panel of Fig 2 depicts the individual search slopes for black and white targets, for both angry and frightened search goals. As is clear from this graph, the search slopes were smaller for black targets than for white targets for each of the two emotions. An increase in set size with 4 faces affected the response times less for black targets (resulting in a search slope of 68 (68) ms per added face) than for white targets (resulting in a search slope of 84 (83) ms per added face), reflected by a significant interaction between Race and Set Size (*F*(1,75) = 6.84, *p* = .01, partial η^2^ = .08 (*F*(1,71) = 5.50, *p* = .02, partial η^2^ = .07)). This indicates that black male faces were detected significantly more efficiently than white male faces.

Interestingly, angry faces were detected more efficiently than frightened faces (Target Emotion* Set Size, *F*(1, 75) = 10.1, *p*< .01(*F*(1,71) = 8.22, *p* = .005, partial η^2^ = .10)). This finding that is in line with previous results showing that angry faces are detected more efficiently than happy faces [21], suggesting that angry facial expressions are processed faster and more efficiently than other emotional expressions.

Importantly, the absence of an interaction between Target Emotion, Set Size and Race (*F*(1, 75) = .03, *p* = .87, partial η^2^ < .001(*F*(1,75) = .01, *p* = .92, partial η^2^ < .001)), makes it clear that the effects of race on attention are present independent of the emotion that people are searching for (and vice versa). So, black targets were detected more efficiently, whether participants were searching for a fearful or angry face (and angry faces were detected more efficiently than frightened faces for both black and white targets).

## Experiment 2

The results of experiment 1 indicate that black faces draw attention when people search for negative emotions such as anger and fear. This attentional focus on black faces could very well be based on the automatic association between ‘black’ and ‘negative’. Here, I explored whether the attentional preference for black faces is indeed related to the strength of one’s Black + Bad associations. I tested this by exploring the relationship between an individual’s implicit anti-black/ pro-white bias, as measured by the Implicit Association Task (IAT, Greenwald, McGhee, & Schwartz, 1998), and racially guided attention for faces.

### Methods

#### Participants

I tested 78 participants (47 women, age range 17–59 years, average 23.5 years) from the Harvard University Subject Pool for payment or course credit. Of these 78 participants, 36 participants were European Americans, 21 were Asian Americans, 11 were African American, 8 were Hispanic Americans and 2 participants did not answer the question.

#### Procedure

The design of Experiment 2 was similar to experiment 1, except that in Experiment 2 the target faces (present in 80 out of 160 trials) were always angry. In addition, both target- and distractor-faces were full color pictures of real faces, taken from the Face Database of the Center for Vital Longevity [22] and the MacBrain Face Stimulus Set (Development of the MacBrain Face Stimulus Set was overseen by Nim Tottenham and supported by the John D. and Catherine T. MacArthur Foundation Research Network on Early Experience and Brain Development. Please contact Nim Tottenham at tott0006@tc.umn.edu for more information concerning the stimulus set). 10 black men and 10 white men, pictured with a neutral and angry expression, were selected. Of these 20 men, 8 had their mouth slightly open in the neutral expression and 9 in the angry expression.

After the search task, participants completed an IAT measuring the relative strength of association between *black-white* and the attributes *good-bad*. In this task, participants were instructed to categorize words and pictures as fast as they could. Pictures were 6 black (3 male, 3 female) and 6 white (3 male, 3 female) faces cropped at forehead and chin, which were to be categorized as ‘black’ or ‘white’. The words, 8 positive (joy, love, peace, wonderful, pleasure, glorious, laughter, happy) and 8 negative (agony, terrible, horrible, nasty, evil, awful, failure, hurt), were to be categorized as ‘good’ or ‘bad’. In addition to 3 practice blocks, participants completed 2 critical blocks (40 trials): a congruent and an incongruent block. In the congruent block, the categories ‘good’ and ‘white’ were to be categorized by one hand, and ‘bad’ and ‘black’ by the other. In the incongruent block ‘good’ and ‘black’ were to be categorized by one hand and ‘bad’ and ‘white’ by the other. The sequence of the incongruent and the congruent blocks as well as the attribution of categories to the left and right hand were counterbalanced over participants.

#### Analyses

Reaction times to trials in which participants correctly identified the presence of the target face were corrected for outliers using the same procedure as in Experiment 1. As a result of the outlier correction and removal of misses 18% of the data were removed (13% based on incorrect responses, 5% following outlier correction). The IAT *D* score was computed based on the algorithm defined by Greenwald, Nosek and Banaji [23]. A positive *D* score indicated that the association between Bad + Black was stronger than the association between Good + Black. To test whether the IAT score was correlated with the overall black-over-white attention bias, I also computed a relative bias score by computing difference between each participant’s search slope for black angry targets and white angry targets (i.e. the slope for white angry targets minus the slope for black angry targets).

To test for differences in search slopes, and whether these differences correlated with the IAT *D* scores, I conducted an ANOVA with Set Size (4/8) and Race of Target (black/white) as within-subjects factors and the IAT *D* scores as a moderator variable.

The results are reported for the full sample of participants (78 participants), as well as for a smaller sample that excludes the African American participants (67 participants). The statistical tests for the full sample are always presented first, with the results from the smaller sample presented in parentheses.

### Results and Discussion

The IAT *D* score ranged from -.84 to .95 with a mean of .21 (.23), indicating that the typical association of black + bad over black + good was present (*t*(77) = 4.53, *p* < .001, Cohen’s *d* = 1.02 (*t*(66) = 4.55, *p* < .001, Cohen’s *d* = 1.12)).

**Fig 3:**
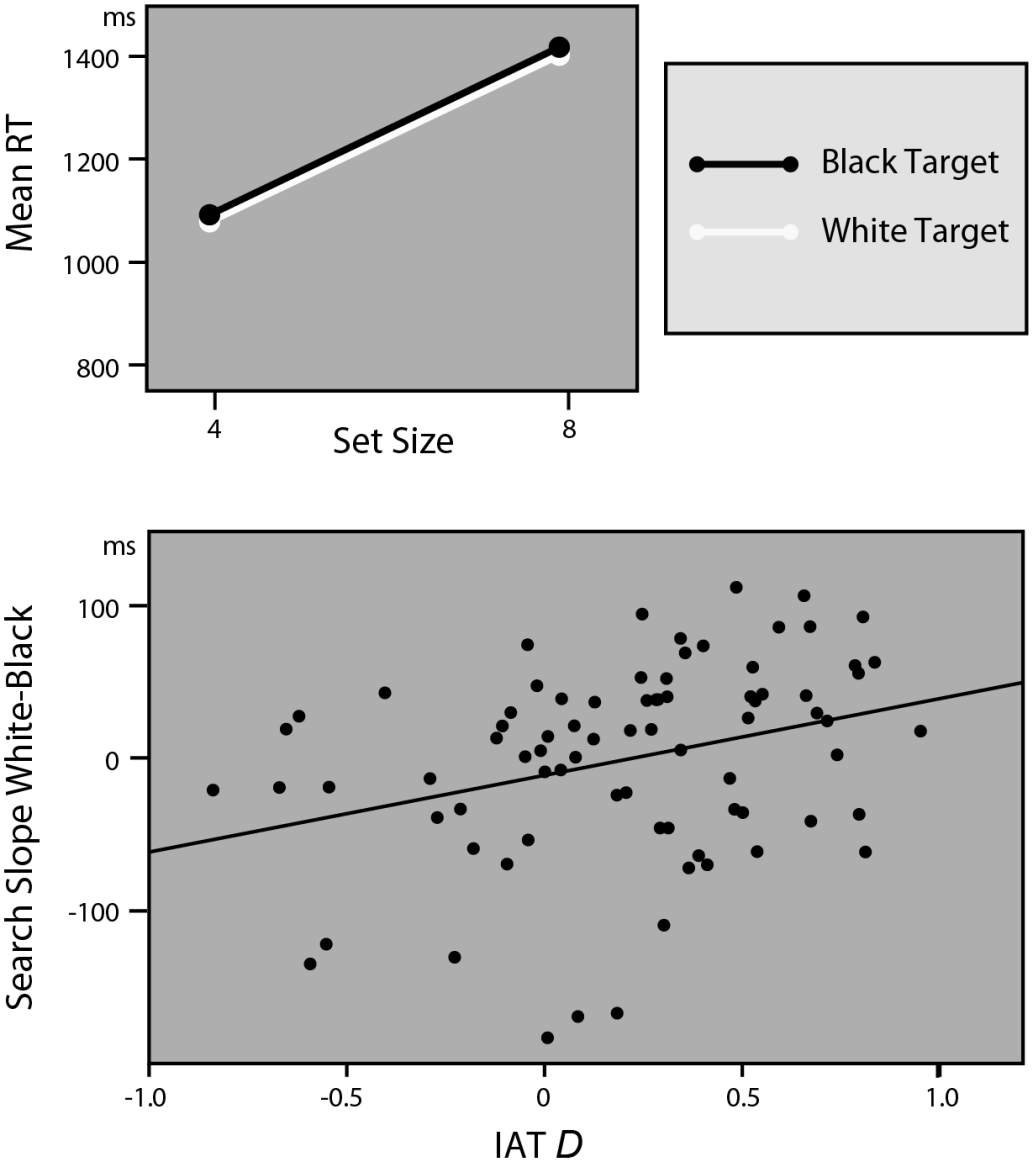
Results of Experiment 2. Angry Targets. Top panel shows the search functions for black and white targets when pictures of people are used as targets and distractors. The search functions are identical, signaling that, over all participants, black faces do not attract more attention than white faces. The bottom panel depicts association between the IAT D scores and the difference between search slopes for black and white targets, showing that people with a stronger anti-black/ pro-white bias (higher IAT D scores) attend to black faces over white faces.

As the top panel of Fig 3 indicates, participant were no faster to detect the angry black target compared to white targets (*F*(1, 77) = .13, *p* = .72, partial η^2^< .01 (*F*(1, 66) = .09, *p* = .77, partial η^2^ < .01)). It did take participants longer to find the angry face in an 8 face display compared to a 4 face display (Set Size, *F*(1, 77) = 344.65, *p*< .0001, partial η^2^ = .82 (*F*(1, 66) = 259.21, *p*< .0001, partial η^2^ = .80)), but this increase in search time was not significantly influenced by the race of the target (Race*Set Size, *F*(1, 77) = .01, *p* = .94, partial η^2^< .001 (*F*(1, 66) = .01, *p* = .94, partial η^2^ < .001)). Indeed, virtually identical search slopes for the two categories (86 (85) ms for black, and 86 (85) ms for white targets) indicate that overall black and white natural male faces are found equally efficiently.

The scatter plot in Fig 3 shows the relationship between each participant’s IAT D score and the difference between each participant’s search slope for black angry targets and white angry targets (i.e. the slope for white angry targets minus the slope for black angry targets). Higher positive IAT D scores indicate a stronger association between the concepts ‘black’ and ‘bad’ compared to ‘black’ and ‘good’. Higher RT differences indicate that search for black targets is more relatively efficient than search for white targets. As is clear from the scatterplot, those people who had a stronger anti-black (or pro-white) bias also showed more efficient search for black men over white men, while those who had stronger anti-white (or pro-black) bias showed a somewhat more efficient search for white over black male faces. This association between a stronger anti-black/ pro-white bias and increased attention for black men (*r* = .32 (*r* = .33)) is corroborated by a significant interaction between Race, Set Size and IAT Scores (*F*(1, 76) = 8.71, *p*< .01, partial η^2^ = .10 (*F*(1, 65) = 7.88, *p* = .007, partial η^2^ = .11)). It thus seems that those participants who have stronger implicit racial stereotypes, focus more of their attention on the faces with which they have negative associations.

The results of Experiment 2 therefore elaborate the findings of Experiment 1, showing that individual (implicit) biases play an important role in the attentional bias for black over white faces, since only those participants with an anti-black/ pro-white bias show this attentional preference for black targets relative to white targets. Those participants with neutral or positive implicit associations for black faces show no attentional bias for black faces, or even a reversed attentional bias for white face (on average, search time increased 96ms per added face for black targets, and 81ms for white targets for those participants in the lower half of the IAT scores). Only those participants with negative implicit associations for black faces showed a clear relative attentional bias for black faces over white faces (on average, search time increased 75ms per added face for black targets, and 91ms for white targets for those participants in the upper half of the IAT scores). These results suggest that the attentional bias is twofold: participants with a strong anti-black/ pro-white bias have a relative attentional bias for black targets, combined with a relative decrease in attention for white faces.

However, the results are different from Experiment 1 in that without taking implicit bias into consideration, black and white faces did not show a difference in attentional processing (as indicated by the non-significant interaction between Race and Setsize). Therefore an independent replication of Experiment 2 has been completed, testing 58 Dutch participants (of Dutch or Middle Eastern descent). The replication showed that black search targets evoked lower search slopes than white targets (TargetRace * Setsize; *F*(1,57) = 3.3; *p* = .075, partial η^2^ = .06). Similar to the findings of Experiment 2, the relative increase in attention for black over white faces was positively correlated to the IAT-d score (r = .258, Race * Setsize * IATD: *F*(1,56) = 3.68; *p* = .06, partial η^2^ = .06). If we collapse Experiment 1 (angry search targets only), Experiment 2 and the replication, the interaction of race and setsize, signaling an attentional bias for black over white faces, is also present (F (1,211) = 6.2; p = .01, partial η^2^ = .03). Taken together, the results of Experiment 1, 2 and the Dutch replication show a reliable attentional bias for black over white faces, which seems to be dependent on implicit racial preferences.

## Experiment 3

Another way to approach the question of the relationship between automatic attitude and attention is to look for discriminant validation. If increased attention to black men found in Experiment 1 and 2 reflects a general increase in attention for the stereotyped group (i.e. black men), then black faces should also attract attention over white faces when the search task requires detection of a happy face among neutral faces. As in Experiment 2, this attentional preference for black faces could be correlated with race bias. If, on the other hand, participants only attend to the less preferred group members when the search goal is a negative emotion such as anger or fear, then black and white men should be attended equally when participants are searching for a positive facial expression, and I should find no correlation between race-based attentional preferences and implicit race bias.

### Methods

#### Participants

I tested 80 participants (51 women, age range 15–59, average 19.2 years) from the Harvard University Subject Pool participated for course credit. Of these 80 participants, 44 participants were European Americans, 23 were Asian Americans, 6 were African American, 6 were Hispanic Americans, and 1 was Hawaiian.

#### Procedure

The procedure and design of experiment 3 was identical to Experiment 2, except that the target faces now had a happy expression. The pictures that were used were of the same black and white men as in Experiment 2, now with a neutral or happy expression.

#### Analysis

Reaction times to trials in which participants correctly identified the presence of the target face were corrected for outliers using the same procedure as employed in Experiment 1. As a result of the outlier correction and removal of misses 12.5% of the data were removed (7.5% as a result of incorrect responses, 5% following outlier correction). The remaining response times were analyzed using an ANOVA with Set Size (4/8) and Race of Target (black/white) as within-subjects factors, and IAT *D* score as a moderator variable.

The results are reported for the full sample of participants (80 participants), as well as for a smaller sample that excludes the African American participants (74 participants). The statistical tests for the full sample are always presented first, with the results from the smaller sample presented in parentheses.

### Results and discussion

The IAT *D* score ranged from -.65 to 1.13 with a mean of .35 (.34), again indicating a stronger association between Black + Bad than Black + Good (*t*(79) = 7.48, *p* < .001, Cohen’s *d* = 1.68 (*t*(73) = 7.00, *p* < .001, Cohen’s *d* = 1.64)).

**Fig 4:**
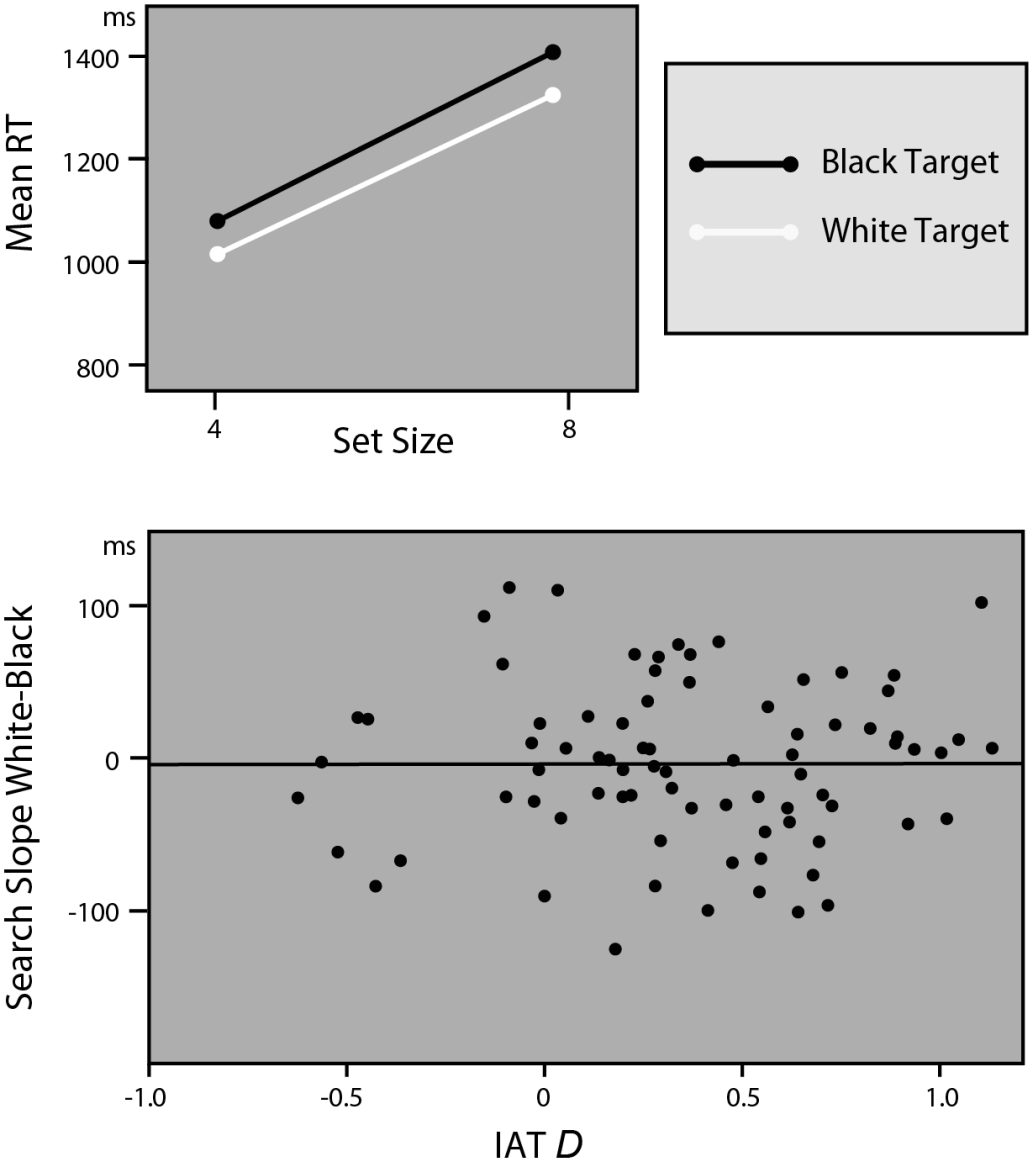
Results of Experiment 3. Happy Targets. Top panel shows the search times per set size for black and white targets. The identical gradients of the lines indicate no attentional preferences for black over white faces. The bottom panel illustrates the absence of any association between the IAT score and attentional preference for black over white faces.

The top panel of Fig 4 shows that Happy white targets were identified faster than black targets (Race, *F*(1,79) = 16.21, *p*< .0001, partial η^2^ = .17 (*F*(1,73) = 18.29, *p* < .0001, partial η^2^ = .20)), and that it took participants longer to identify a target in an 8-face display than in a 4-face display (Set Size, *F*(1,79) = 293.32, *p*< .0001, partial η^2^ = .79 (*F*(1,73) = 255.49, *p* < .0001, partial η^2^ = .78)). However, the absence of a difference in search slopes for black and white targets (Race*Set Size, *F*(1,79) = .22, *p* = .64, partial η^2^ < .01 (*F*(1,73) = .13, *p* = .73, partial η^2^ = .002), search slope is 82 (82) ms for black and 80 (80) ms for white targets) indicated that neither black nor white men attracted more attention over participants. In addition, it is also clear from the scatterplot in Fig 4 that racial bias did not correlate with relative search efficiency for black vs. white happy faces (*r* = .02 (*r* = .006), *F*(1.78) = .03, *p* = .86, partial η^2^ < .001 (*F*(1,72) = .003, *p* = .96, partial η^2^ < .0001)). When people are instructed to search for a positive target, black men do not attract attention over white men, not even for those participants who have a strong anti-black/ pro-white bias.

## General Discussion

When someone searches a crowd for people that might cause trouble, will their attention be guided to black men, and decrease for white men? The outcome of the present set of experiments suggests this will be the case, especially so if that person has a relatively strong automatic negative attitude toward black people. However, the relative attentional preference for black compared to white men is only present when the task involves a search for anger or fear. So, in the searching for a happy face, black and white men will be attended to equally.

Several studies have shown that implicit intergroup attitudes influence behavior towards individual group members (for a review see [24]). Here I show that automatic racial attitudes play a role in the very first stages of social information processing. The current results suggest that race preferences not only change our actions [25,26] and evaluations [27], but also the manner in which one attends to the world. Importantly, the current set of studies show that these attentional effects of race are present even when participants are not explicitly searching for faces with specific (black or white) racial features [16,17]. This indicates that racial information is used to guide attention automatically, independent of whether race is a relevant factor in the task or not.

The fact that attention is only guided by race when people are searching for a negative emotion might be related to the fact that anger is stereotypically associated with black men (Devine, 1989), which was also the main reason why I only included male search targets in our experiment. The search goal (i.e. anger in Experiment 1 and 2) could have functioned as an orientation signal, guiding attention to the prime-related black faces (see also [28]). This idea is consistent with the fact that I observed no increased attention for black faces in Experiment 3, where participants searched for happy faces. However, in Experiment 1 participants showed an increase in attention for black faces independent of whether they were trying to detect an angry or a frightened face. This suggests that the attentional preference is not so much based on the cultural stereotype of the “angry black man”, but more on the (unconscious) expectation that people with whom one has negative associations are more likely to show a negative emotion. Following the line of reasoning that negative implicit associations guide the attentional focus in case of a negative search goal, not just anger or fear but all negative emotional search goals (e.g. sadness, humiliation, shame etc.) should result in an attentional preference for black faces. Obviously, the current data set is suggestive with regards to this idea, but by no means conclusive, as I only explored anger (Experiment 1 &2) and fear (Experiment 1). It would also be interesting to test whether this association between black and negative emotions is specific for male targets, or whether it is also present for female faces.

It is also possible that the attentional bias for black faces when people are searching for anger is related to other findings from the emotion perception literature, showing that people are faster to perceive anger on the face of an African American than of a European American [29]. If these results are based on the same underlying process as our current findings, this suggests that attention is not just attuned to black faces when people are searching for anger: Hugenberg and Bodenhausen’s findings would suggest that the attentional spotlight is also particularly attuned to anger when participants are looking at a black face.

The current set of results do not suggest that outgroup faces automatically attract attention, they do not ‘pop out’: Outgroup faces only attract attention for those people who have negative associations with that particular outgroup and are searching for a negative feature. Interestingly, some research has shown that group-membership in itself, independent of search-goals and negative traits associated with specific groups, is enough to guide visual attention. Brosch and Van Bavel [30] showed that a one-minute instruction session to indicate which faces belonged to the outgroup and which faces belonged to the ingroup, was enough for those faces associated with the outgroup to consistently draw attention in a dot-probe task. Obviously, in such a minimal group paradigm, no threat is associated with the new and undefined outgroup. As such, the attentional focus on the outgroup cannot be explained by underlying explicit negative associations with the outgroup. In a similar vein, Al-Janabi and colleagues [31]showed that faces belonging to a racial outgroup that is not associated with threat, i.e., East-Asian faces, still evoked stronger automatic capture of attention in a dot-probe task. Together, these experiments suggest that people automatically focus their attention on outgroup faces. One reasons why the current set of experiments find that attentional focus on outgroup faces is a bit more complicated than this could lie in the type of task that was used. The current experiments all employ a visual search task which asks people to locate a specific face in a relatively crowded search display. The studies that found automatic attention to outgroup faces all employed a dot-probe task, which employs displays with only two faces present at a time. It is possible that in a sparse visual environment, group membership in itself is more easily detected, and therefore more automatically used, whereas in a more complicated visual environment group membership is only used to guide attention when it is internally (based on prejudice) or externally (based on the search-goal) relevant. This hypothesis, however, should be confirmed by future research.

One could argue that the differences in attention for black and white targets found in Experiment 1 and 2 can be explained by differences in low-level visual features between black and white faces. This would mean that attention is not drawn to black faces because of their race, but because in these specific sets of stimuli, black faces differ from white faces in, for example, contrast [32]. However, the current set of experiments suggests that I cannot reduce the attentional preference for black faces to differences in basic visual features. First of all, I have replicated the race-based attentional preference over two different sets of stimuli, using computer-generated faces in Experiment 1 and real faces in Experiment 2. Secondly, in Experiment 2 I show that the attentional preference for black faces is related to individual differences in automatic race bias. Thirdly, the effects of race on attention disappear when the search goal is changed, as is the case in Experiment 3, while the basic racial features of the stimuli remain similar to the stimuli used in Experiment 2. Overall, this strongly suggests that the observed differences in attention are not due to differences in low-level visual features, but truly reflect higher-level search goals and cognitions about race.

## Conclusion

In 3 experiments I showed that, when people are searching for negative social targets, such as anger or fear, their attention is guided by their group-based social attitudes. Since attention is considered the gateway to conscious perception (Posner, 1994) these findings suggest that our automatic social evaluations along a good-bad dimension can alter not only what or who catches our eye, but also how we perceive our environment. The influence that racial attitudes have on attention and on perception could be one of the reasons why racial stereotypes are so pervasive: it makes us more likely to see those things that we believe to be true.
